# Equivalences in Biological and Economical Systems: Peloton Dynamics and the Rebound Effect

**DOI:** 10.1371/journal.pone.0155395

**Published:** 2016-05-12

**Authors:** Hugh Trenchard, Matjaz Perc

**Affiliations:** 1 Independent researcher, Victoria, Canada; 2 Faculty of Natural Sciences and Mathematics, University of Maribor, Koroška cesta 160, SI-2000 Maribor, Slovenia; 3 CAMTP—Center for Applied Mathematics and Theoretical Physics, University of Maribor, Krekova 2, SI-2000 Maribor, Slovenia; National Scientific and Technical Research Council (CONICET)., ARGENTINA

## Abstract

An interdisciplinary bridge is proposed between principles of collective behavior in biological systems, particularly bicycle pelotons, and the economic phenomenon called the rebound effect. Two main equivalencies are proposed between aspects of peloton dynamics and aspects of energy service efficiencies and the rebound effect. Firstly, a threshold whereby weaker cyclists, up to maximal capacities, sustain speeds of pacesetters by drafting; equivalent to a threshold whereby consumers will not exceed maximum allocated budgets for energy services, costs for which are externally determined. Secondly, a threshold of peloton dynamics whereby, below this threshold, weaker cyclists share costly non-drafting positions, whereas above this threshold cyclists cannot share these positions but can sustain pacesetter speeds. This is in turn equivalent to the threshold in the context of energy service efficiency, whereby consumers will increase spending to the limit indicated by the rebound magnitude but not to their maximum allocated budgets. These thresholds are a consequence of the model equations, and the latter threshold is explained by consumer apprehension that existing energy efficiencies could disappear or be negative, when consumers would be over budget. This partly explains long term rebound increase, whereby consumers increase consumption as confidence rises that cost savings due to energy service efficiency is stable.

## Introduction

In any social, political, or institutional setting, leaders facilitate energy savings for followers by giving direction and increasing efficiencies in energy expenditure. Such efficiencies are achieved by preventing mistakes or effort spent on problems that have established solutions. Similarly, teachers save pupils from wasting energy for much the same reasons. Perhaps more tangibly, forms of energy savings occur in a variety of other biological [1–4] and even some non-biological systems [5], whereby leading agents physically pave the way for followers who thus obtain reductions in their own energy expenditures. These systems are examples of complex systems, and only relatively recently has economics been recognized as susceptible to the modelling approaches of complexity science [6,7].

Among biological systems in which followers experience reduced energetic expenditure, are bicycle pelotons (groups of cyclists) [8]. Cyclists in pelotons save energy by drafting, or following in zones of reduced air resistance [8]. As cyclists adjust their speeds, pelotons exhibit phases of collective behavior that oscillate between compact, high density, formations, and elongated, single-file formations [9].

Where are the energy savings mechanisms in economic systems? Businesses, for example, may reduce costs by replicating the successful practices of others, or engage in cooperative dynamics that reduce the costs associated with competitive strategies [10]. Relatively recent investigations into the benefits of corporate cooperation include [11, 12]. One economic condition well-studied quantitatively is the rebound effect and its relation to improved energy efficiencies [13–15], which we argue is another example of an economic energy savings mechanism. Broadly, the rebound effect occurs when increasing the efficiency in delivery of certain energy-related services results in some “take-back” or increased consumption of those services [13, 14].

We propose a model of the rebound effect based on peloton dynamics, wherein the energy savings mechanism is a fundamental component of the model. The model represents an interdisciplinary bridge whereby energetic costs in a biological sense are equated to costs in an economic sense. In this way, we identify a narrow set of common principles between biological systems and economic systems that point to some underlying universal principles or properties that govern these two seemingly disparate systems. While the attempt to unify biological and economic principles is not new [16], the proposed model deepens the interdisciplinary connection and permits predictions for observations of collective behavior both in the abstract sense of the “invisible hand” of economics, as well as in respect of the behavior of individual consumers.

## Methods

Rebound occurs in three main conditions:

1. A direct effect in which the cost of a given energy service falls as that service undergoes increased efficiency. This results in a direct increase in consumer consumption of that service, which offsets the expected magnitude of the reduced energy services consumption;
2. An indirect effect in which the improved energy service and corresponding decreased costs results in increased consumption that is distributed across other energy-requiring services;
3. Economy-wide rebound that is the sum of direct and indirect effects [13, 14].

Sorrell and Dimitropolous quantify the magnitude of energy savings and rebound [13,14]:

ηε(E)=ηε(S)−1 (1)

where *ŋ*_*ɛ*_(*E*) is the elasticity of demand for energy (*E*) with respect to energy efficiency (ɛ); *ŋ*_*ɛ*_ (*S*) is elasticity of demand for services (*S*) with respect to energy efficiency. The actual saving in energy consumption equals empirically predicted savings when *ŋ*_*ɛ*_(*S*) = 0, and the reduction in energy consumption due to the improved efficiency in the energy service (i.e. demand for energy services) is *ŋ*_*ɛ*_(*E*) = -1 [13]. In [14], Sorrell et al. state the rebound effect is usually given in percentages and they give the example where *ŋ*_*ɛ*_ (*S*) = 0.2 to yield a value for *ŋ*_*ɛ*_(*E*) of -0.8, such that a 20% rebound means that 0.2 is “taken back” from the -1 total reduction in consumption due to the improved service, for a value for *ŋ*_*ɛ*_(*E*) of -0.8.

Here we introduce an alternative version of Eq 1, whereby total energy service cost savings is:

$$E_{t}=E-(E*r)$$ (2)

where *E*_*t*_ is the total actual energy savings; *E* is the expected energy service savings, and *r* is the rebound, both expressed as percentages or fractions of 1. Thus a 20% rebound of a 50% expected energy savings produces *E*_*t*_ *=* 0.4, or 40% actual energy savings. Here -1 in Eq 1 is replaced by the actual percentage savings due to the improvement in services; e.g. -1 is equivalent to 50% energy savings, multiplied by 0.8, for 40% total energy savings (*E*_*t*_). Eq 2 has the advantage of quantifying very simply the total actual energy savings in percentage terms after the rebound magnitude is accounted for, while Eq 1 yields the coefficient to be applied against whatever the expected savings happens to be without necessarily accounting for the expected savings quantity.

It turns out that Eq 2 has a precise analogue to a critical term in a fundamental equation that describes bicycle peloton dynamics, where the coupled relationship between the leader and follower is described:

$$PCR=\frac{P_{front}*d}{MSO_{follow}}$$ (3)

where PCR is the “peloton convergence ratio”, *P*_*front*_ is the power output of the pacesetting rider at the given speed, *d* is the coefficient of drafting; i.e. the ratio of the required power output of the drafting rider to the power output of the front, non-drafting rider, and *MSO*_*follow*_ is the maximal sustainable power output of the following rider.

Adapting this fundamental equation of peloton dynamics in terms of the rebound effect we get the equation:

$$R=\frac{I\;*\;E_{coeff}}{MSC}\;,$$ (4)

for the “rebound ratio”, describing the combined impact of the rebound and energy savings on individual consumers as a proportion of their spending capacity. *I* is the initial cost, in monetary terms, of the service prior to the introduction of the energy service efficiency, externally imposed on the consumer (akin to a pacesetter in cycling, who imposes the speed to be sustained by the following rider). As stated, *E*_*t*_ is the total energy service savings expressed as a percent or fraction of 1; *E* is the expected energy efficiency, and *r* is the rebound, both expressed as percentages, or fractions of 1. *MSC* is a consumer's maximal spending capacity. *E*_*coeff*_ is 1-*E*_*t*_, equivalent to *d* in pelotons, which is a ratio of the output of the following rider in the drafting position to the output of the non-drafting front rider. As stated, *E*_*t*_ = *E–*(E* r) and the complete equation is:

$$R=\frac{I\;*[1-(E-(E\;*\;r))]}{MSC}.$$ (5)

## Results and Discussion

Eq (5) describes the coupled relationship between the initial cost of the energy service (≡ pacesetter cyclist in a peloton) and consumer’s income (≡ drafting cyclist whose energetic costs are reduced by the energy savings of drafting), whose effective spending capacity increases when a given energy service efficiency is introduced. Just as a cyclist increases her effective maximal speed by drafting a pacesetter, so does a consumer’s available income increase as a result of the improved energy efficiency and the effectively reduced cost of the given energy service. This increased efficiency permits some “take-back” of speed or increase in consumption, as the case may be. The threshold *R* = 1 indicates the point when the cost of the service de-couples from capacity of the consumer to pay for it, just as where *PCR* = 1 indicates the point when a following cyclist can no longer sustain the pacesetter’s speed.

**Fig 1:**
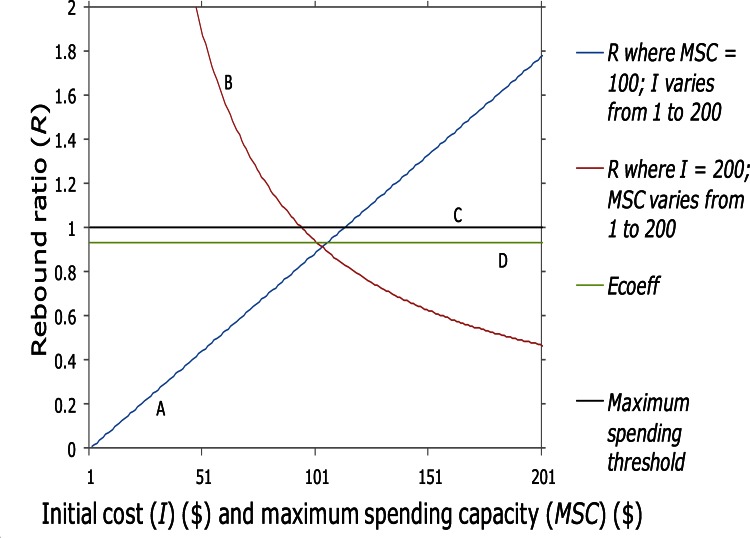
Rebound ratio curves. To illustrate, for curve A the initial cost (*I*) of the energy service varies between $1 and $200; for curve B a consumer’s maximum spending capacity (*MSC*) varies between $1 and $200. Curve C is the de-coupling threshold between the cost of the service as attenuated by the energy services savings quantity, and the consumer’s capacity to pay. The energy service efficiency is 0.08 based on [17], and the rebound is 0.22, based on data in [14], and *E*_*coeff*_ is 0.9376 (curve D).

Further, *R* allows us to predict consumer behavior in response to the value of *E*_*coeff*_: If *R* < 1 for a consumer, that consumer may increase consumption of the given energy service and incur its corresponding marginal cost as a proportion of his or her *MSC*, up the maximum point when *R* = 1 (curve C in Fig 1).

**Fig 2:**
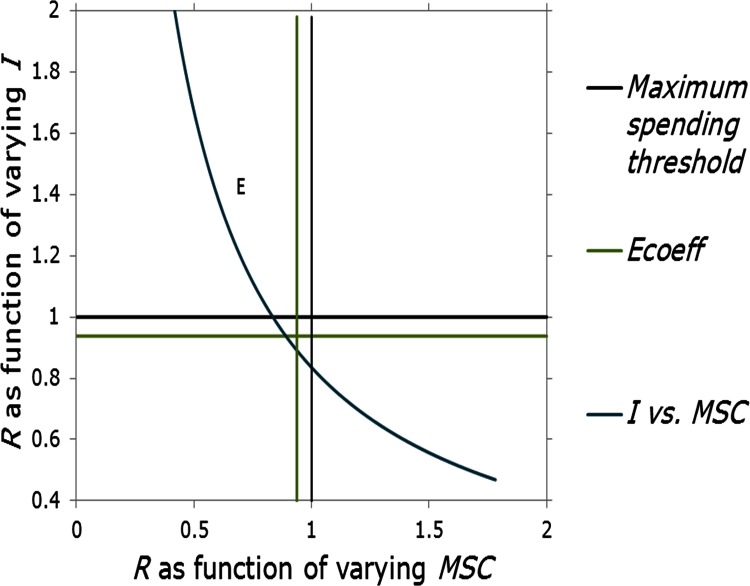
Rebound ratios as function of varying the initial cost (*I*) and the maximal spending capacity (*MSC*). Parameter values for vertical and horizontal curves are as in Fig 1.

A consumer’s consumption of the energy service necessarily must decrease when *R* > 1. If the cost (*I*) of the energy service increases such that R > 1, a consumer will need to lower his *MSC* so as not to exceed *R*. The inverse relationship between *I* and *MSC* is shown in Fig 2 (curve E).

However, many consumers may be uncomfortable with the prospect of maximizing budget expenditure for a given service, particularly if they face the prospect of maximizing total household income for the various goods and services they require and are not able to transfer income from one service to another. This implies that there is some consumer spending threshold that is lower than *R* = 1.

Peloton theory offers insight into where this threshold lies, which by analogy suggests this threshold is approximately *R* = *E*_*coeff*_, shown as curve D in Fig 1, and shown in Fig 2. This is analogous to the “protocooperative threshold” in pelotons (PCR = *d*) [18]. When above this threshold, following cyclists can maintain the speeds of pacesetters at some output less than *MSO*, but cannot accelerate without the drafting benefit of the pacesetter; i.e. if cyclist X is drafting at one instant but then pulls to the side, outside drafting range of the leader Y, X is instantaneously at *MSO*, and cannot pass rider Y [18]. When cyclists are below the protocooperative threshold, they are capable of passing behavior and naturally engage in cooperative behavior because they have sufficient surplus energy to share the most costly leading positions [18].

Thus in Fig 1, for curve A, if *MSC* is constant and *I* varies, if *I* exceeds $100 then it exceeds the consumer’s capacity to afford it. At the intersection of A-D, the consumer can in principle afford to increase paying *I* up to the intersection of curve A-C, as long as the energy service savings is present. However, in practice we suggest that the consumer is unlikely to continue to increase paying for cost *I* in the region between curves D and C, because any further increased spending by the consumer that fills the new effective spending space permitted by the energy savings quantity (*E*_*t*_), means that if for some reason the energy savings disappears (or if *I* increases), our consumer is suddenly over-budget (i.e. *R* > 1).

We emphasize that the prediction that consumers tend not to exceed the *R*_*coeff*_ threshold (curve D) is fundamentally a consequence of Eq 2 and the presence of the energy savings quantity. This is an observable and demonstrable physical phenomenon of peloton dynamics whereby a following cyclist, approaching his maximal capacity while in a drafting position, who suddenly finds himself in a position without the drafting benefit, instantaneously exceeds his maximal capacity and is forced to markedly decelerate. However, considerable further work will be needed to establish evidence for this proposition in an economic context. Such evidence should include the relative duration and stability of any energy savings quantity and other factors that tend to cause fluctuations in intial cost (*I*).

For curve B, tracing upward from right to left, if *I* is constant and a consumer’s spending capacity varies, a consumer’s *MSC* cannot fall below the intersection of curves B-C, and as described above is unlikely to pay the value of *I* in the region between D and C, at least until the energy savings quantity and *I* are stable.

### A simple example

Consumer *x* has a maximum budget for household heating of $100/month (*MSC*). Initially, the heating service cost is $100/month (*I*). New technology improves the service, and reduces *x*’s heating bill by $40/month (*E* = 0.4, and *E*_*coeff*_ = 1–0.4).

$$\begin{array}{l} R=\frac{I\;*\;E_{coeff}}{MSC}\\ =\$100\;*\;0.60\;/\;MSC\\ =0.60 \end{array}$$

Short run rebound is 20%, meaning *x* increases consumption of the $40 saving by 20% (*r* = 0.2), and so *x* spends an additional $8.00/month on heat, for a total of $68/month.

$$\begin{array}{l} R=\frac{I\;*\;E_{coeff}}{MSC}\\ \;=\$100\;*\;0.68\;/\;\$100\\ \;=0.68. \end{array}$$

Here *E*_*coeff*_ is 1- *E*_*t*_ where *E*_*t*_ = *E–*(E* r).

In this scenario, if *E* falls suddenly to 0 (along with any rebound) and the energy service cost is simply *I* (i.e. $100), then *R* = 1; *x* therefore spends his maximum on the service. If there is some excess *reduction* in efficiency such that *E*_*coeff*_ > 1, then *R* > 1, and *x* is forced to reduce service consumption, increase his *MSC* somehow, perhaps by improved employment, or by borrowing funds to finance the increase in services, as shown in Fig 2.

However, if *E* remains stable for two years, *x* becomes more comfortable with the notion of increasing his use of the service and incurring slightly higher cost. Thus the long term rebound increases to, say, 30% (*r* = 0.30) (slope D in Fig 1 shifts upward relative to *R* = 0.68 in previous example).

$$\begin{array}{l} R=\frac{I\;*\;E_{coeff}}{MSC}\\ \;=\$100\;*\;0.72\;/\;\$100\\ \;=0.72 \end{array}$$

Now if *x*’s heating budget (*MSC*) increases to $200 and the other original values stay the same with no rebound, *R* = 0.30, and with rebound, *R* = 0.36.

## Conclusions

Three basic parameters of peloton dynamics analogous to the rebound effect are: (i) The speed or output established by a leader or pacesetter, which corresponds to the initial cost to the consumer of the energy service provided, externally established (i.e. external to the consumer); (ii) The energy savings permitted by the energy savings mechanism, which corresponds to the increase in energy efficiency due to some improvement in technology that reduces the cost of energy service delivery; and (iii) The *MSO* of a follower who seeks to maintain the pace set by the pacesetter, which corresponds to the individual consumers’ maximum income allocated to the energy service.

Obtaining Eq (5) as the analogous equation to the peloton model [18, 19], the following broad aggregate effects, or dynamical phases, are proposed in the context of energy service efficiencies and rebound effects:

- A relatively high collective rebound effect that indicates some general economic abundance or surplus (high income relative to consumption);
- A relatively low collective rebound effect that indicates generally strained economic conditions (low income relative to consumption), whereby households have little or no surplus income to contribute to increased consumption of the given energy service or toward substituting surplus income for other goods and services.

These collective phases lead to the proposition that high collective rebound should generally reflect relative economic prosperity (i.e. high consumer wealth), while low collective rebound tends to reflect a strained economy (i.e. low consumer wealth). This proposition is inconsistent with Small and Van Dender’s theory indicating that rebound declines over time as incomes increase [20], whereby an improvement in energy efficiency should have smaller relative impact on the cost of the energy service for higher income groups [14]. Contrary to what we propose, Small and Van Dender’s approach [20] suggests that low rebound should reflect a prosperous economy, or widespread high income. Our contrary proposition in which high rebound reflects relative economic prosperity finds support from [14] who questions the validity of Small and Van Dender’s [20] model and who indicates that it is not supported by the meta-data of Hanly et al. [21].

Nonetheless, Small and Dender’s conclusion [20], which implies that energy services costs do not scale across different income levels, indicates our proposed model should be limited to situations in which consumers spend on energy services in approximate proportion to income. Thus a low-income household may incur costs to heat a modest two bedroom bungalow proportionate to household income, while a high-income household may incur costs to heat a ten-bedroom estate: in both cases proportionate heating costs are roughly equal.

Thus we propose that consumer household income, or at least the portion of total household income that consumers are willing to allocate to energy services, is equivalent to the maximal sustainable outputs (*MSO*) of biological agents. All living organisms possess metabolic energy requirements that fall within a range of MSOs unique to each species [22]. The *MSO* of individual cyclists is a fundamental parameter of the collective behavior of bicycle pelotons and, we suggest, other biological systems [18, 19].

Where aggregate *R* values are *R*_*a*_, we propose that, similar to pelotons, when *R*_*a*_ < *E*_*coeff*_, consumers generally hold surplus income, sufficient either to increase spending until *R*_*a*_ = *E*_*coeff*_ or to substitute their surplus income for other goods and services. When *R*_*a*_ > *E*_*coeff*_, consumers still hold some surplus as long as *E* is sustained and in principle can increase spending until *R* = 1 (the region between slopes D and C in Fig 1, and as shown in Fig 2); but if *E* drops for some reason, consumers may no longer have surplus; i.e. since *R >*1 or approaches 1, and so may be unwilling to increase spending until *R* = 1 even while *E* is present. This may also partly explain why short term rebound magnitudes tend to be smaller than long term rebound magnitudes, as is well established by research (mean short run of 21.6% (23 studies) versus mean long run of 27.5% (21 studies)) [14]. Rebound thus increases in the long run as consumers’ confidence increases that cost efficiencies are stable. In this way *R*_*a*_ = *E*_*coeff*_ represents a threshold between broad economic phases of relative prosperity and restraint.

To conclude, our objective is to establish an interdisciplinary bridge between principles of collective behavior in biological systems, and in particular those of bicycle pelotons. This bridge indicates certain universal principles among biological systems and economics.

We demonstrate that there are reasonable equivalences between aspects of peloton dynamics and aspects of economic theory related to energy services efficiency and the rebound effect. In particular, there are two collective behavioral thresholds in peloton dynamics that have reasonable theoretical economic analogues: first, where consumers will not exceed their maximum budgets for energy services, specifically in the context of costs savings due to new energy efficiencies and any rebound increase in spending. A second, lower threshold below consumers’ maximum budgets, is proposed in which consumers will tend not to exceed due to the anticipated possibility that existing energy efficiencies could disappear or be negative, meaning that consumers would then be over budget. This also implies an explanation for long term increases in rebound magnitude, whereby consumers’ confidence tends to increase as the energy efficiency or costs savings is stable, such that consumers tend to increase their consumption of the services nearer to their maximum budget. Further research is required to establish evidence for this proposition.

These proposed analogous principles as between peloton dynamics and economic dynamics, point to deeper common energetic principles that are expressed in biological systems as metabolic outputs, and in economic systems as monetary equivalencies of work outputs. Economic systems are obviously far more complicated than collections of cyclists, birds, fish, or penguins in terms of the factors that determine value and exchange of goods and services, but here at least we demonstrate where some common energetic principles may be found, pointing to fundamental coupling principles common to disparate economic and biological systems, and to novel avenues of interesting research.
